# The BCL-2 pathway preserves mammalian genome integrity by eliminating recombination-defective oocytes

**DOI:** 10.1038/s41467-020-16441-z

**Published:** 2020-05-25

**Authors:** Elias ElInati, Agata P. Zielinska, Afshan McCarthy, Nada Kubikova, Valdone Maciulyte, Shantha Mahadevaiah, Mahesh N. Sangrithi, Obah Ojarikre, Dagan Wells, Kathy K. Niakan, Melina Schuh, James M. A. Turner

**Affiliations:** 1 Sex Chromosome Biology Laboratory, The Francis Crick Institute, London, NW1 1AT UK; 2 Max Planck Institute for Biophysical Chemistry, Am Fassberg 11, Göttingen, 37077 Germany; 3 Human Embryo and Stem Cell Laboratory, The Francis Crick Institute, London, NW1 1AT UK; 4 Nuffield Department of Women’s and Reproductive Health, John Radcliffe Hospital, University of Oxford, Oxford, OX3 9DU UK; 5 IVI-RMA, Magdalen Centre, Oxford Science Park, Oxford, OX4 4GA UK; 6 Duke-NUS Graduate Medical School, Singapore, 119077 Singapore; 7 Department of Reproductive Medicine, KK Women’s and Children’s Hospital, Singapore, 229899 Singapore

**Keywords:** Developmental biology, Embryogenesis, Germline development, Oogenesis, Genetics

## Abstract

DNA double-strand breaks (DSBs) are toxic to mammalian cells. However, during meiosis, more than 200 DSBs are generated deliberately, to ensure reciprocal recombination and orderly segregation of homologous chromosomes. If left unrepaired, meiotic DSBs can cause aneuploidy in gametes and compromise viability in offspring. Oocytes in which DSBs persist are therefore eliminated by the DNA-damage checkpoint. Here we show that the DNA-damage checkpoint eliminates oocytes via the pro-apoptotic BCL-2 pathway members *Puma*, *Noxa* and *Bax*. Deletion of these factors prevents oocyte elimination in recombination-repair mutants, even when the abundance of unresolved DSBs is high. Remarkably, surviving oocytes can extrude a polar body and be fertilised, despite chaotic chromosome segregation at the first meiotic division. Our findings raise the possibility that allelic variants of the BCL-2 pathway could influence the risk of embryonic aneuploidy.

## Abstract (web summary)

If left unrepaired, meiotic DSBs are toxic to mammalian cells, thus oocytes in which DSBs persist are eliminated by the DNA-damage checkpoint. Here the authors provide insights into the roles of PUMA, NOXA and BAX during DNA damage checkpoint that eliminates *Dmc1*^−/−^ and *Msh5*^−/−^ oocytes.

## Introduction

A defining feature of sexual reproduction is meiosis, when paternal and maternal homologous chromosomes synapse and recombine. In mammals, both processes are dependent on meiotic DNA double-strand breaks (DSBs) generated by the SPO11 enzyme^1–3^. DSBs are subsequently processed by the recombination machinery, which includes the RecA homolog DMC1^4,5^ and mismatch repair protein MSH5^6,7^, to form crossovers or non-crossovers^8^. Crossovers allow the homologous pair to be recognised as a single entity by spindle microtubules, and hence facilitate accurate chromosome segregation. Defects in meiotic chromosome segregation produce aneuploid gametes and are the leading cause of congenital disorders and pregnancy loss^9,10^. Chromosome segregation errors arise more frequently and are more likely to remain undetected in the female than the male germline^9,10^. Understanding the pathways that eliminate defective oocytes could permit the development of therapies that enhance fertility in women, by improving the quality of the oocyte pool present at ovulation^11,12^.

Historically, oocyte elimination in mice has been attributed to three quality control mechanisms. Meiotic silencing, the inactivation of genes on asynapsed chromosomes, triggers oocyte loss when one or two chromosomes are asynapsed^13^. A second mechanism, the synapsis checkpoint, triggers oocyte loss when asynapsis is more extensive, and operates even when programmed DSBs are not formed, e.g., in *Spo11*^−/−^ females^14–16^. HORMAD1 and HORMAD2 are implicated in these first two quality control pathways^15,16^. The third mechanism, the DNA-damage checkpoint, eliminates oocytes with persistent DNA damage. Oocytes with defective recombination repair, e.g., *Dmc1*^−/−^^4,5^ and *Msh5*^−/−^^6,7^, exhibit both persistent DNA-damage and chromosome asynapsis, and thus may be eliminated by the combined effects of the synapsis and DNA-damage checkpoint. Several components of the DNA-damage checkpoint have now been identified: repair of lingering DSBs is impeded by the SUMO ligase RNF212, leading to the activation of the checkpoint protein CHK2 and thereafter p53 and p63^17,18^.

Interestingly, recent data has shown that *Spo11*^−/−^ females exhibit spontaneous DNA damage^19^ despite no programmed meiotic DSBs being formed^2,3^. Elimination of *Spo11*^−/−^ oocytes also requires RNF212 and CHK2^18^. Furthermore, the ablation of HORMAD2 reduces the abundance of DNA-damage markers in *Spo11*^−/−^ oocytes^18^. These findings call into question the existence of the synapsis checkpoint, and suggest that the DNA-damage checkpoint may eliminate oocytes in mutants lacking programmed DSB-formation. A prediction of this hypothesis is that the deletion of other checkpoint effectors should rescue oocyte elimination both in mutants with persistent meiotic DSBs, e.g., *Dmc1*^−/−^ and *Msh5*^−/−^ females, and those lacking programmed DSBs, e.g., *Spo11*^−/−^ females.

To date, the checkpoint factors acting downstream of CHK2/p53/p63 to trigger oocyte apoptosis have not been identified. The p53 and p63 transcription factors bind many genomic sites and regulate multiple cellular responses^20^. Among their mitotic targets are intrinsic apoptosis pathway components PUMA, NOXA, and BAX^20^. PUMA and NOXA inactivate members of the pro-survival BCL-2 family, which in turn relieves inhibition of pro-apoptotic factors BAX and BAK and promotes cell death^21,22^. PUMA may also directly bind and activate BAX/BAK^23^. PUMA and NOXA mediate oocyte loss in response to postnatal, γ-irradiation (IR)-induced DSBs^24^, but whether they do so in response to lingering SPO11-catalysed DSBs is not known. Establishing the extent of similarity between exogenous and meiotically-programmed DNA damage responses is clinically important, because it will reveal whether therapies that protect germ cells from genotoxic agents could be useful for the treatment of meiotic infertility. BAX is required for oocyte elimination during development^25–27^ and in response to chemotherapy^28^. However, its role in oocyte loss following IR-induced DSBs or persistent meiotic DNA damage has not been defined.

Here we demonstrate that PUMA, NOXA, and BAX are components of the DNA-damage checkpoint that eliminates *Dmc1*^−/−^ and *Msh5*^−/−^ oocytes. The deletion of BCL-2 components does not rescue oocyte loss in *Spo11*^−/−^ females, demonstrating that the oocyte quality controls operating in these models are genetically dissociable. We also determine the effects of disabling the DNA-damage on oocyte chromosome segregation, maturation, and embryo development.

## Results

### *Puma* and *Noxa* mediate oocyte loss in *Dmc1* and *Msh5* nulls

Exposure to 0.45 Gy of IR at postnatal day 5 (P5) results in the elimination of all primordial follicles by P10 and subsequent infertility in wild-type female mice^29,30^. The same effect is observed in *Noxa*^−/−^ females^24^. However, in *Puma*^−/−^ and *Puma*^−/−^ *Noxa*^−/−^ females, 16% and 52% of oocytes are protected from IR-induced elimination, respectively, relative to non-irradiated mice of the same genotypes^24^. Irradiated *Puma*^−/−^ *Noxa*^−/−^ females are fertile^24^, demonstrating that some oocytes survive beyond P10. In line with these published observations, we found that while primordial follicles were absent in irradiated *Puma*^+/−^ *Noxa*^+/−^ controls at P21, 30% of primordial follicles survived elimination in irradiated *Puma*^−/−^ *Noxa*^−/−^ females (Supplementary Fig. 1a). These findings support the involvement of *Puma* and *Noxa* in IR-induced oocyte loss^24^.

**Fig. 1:**
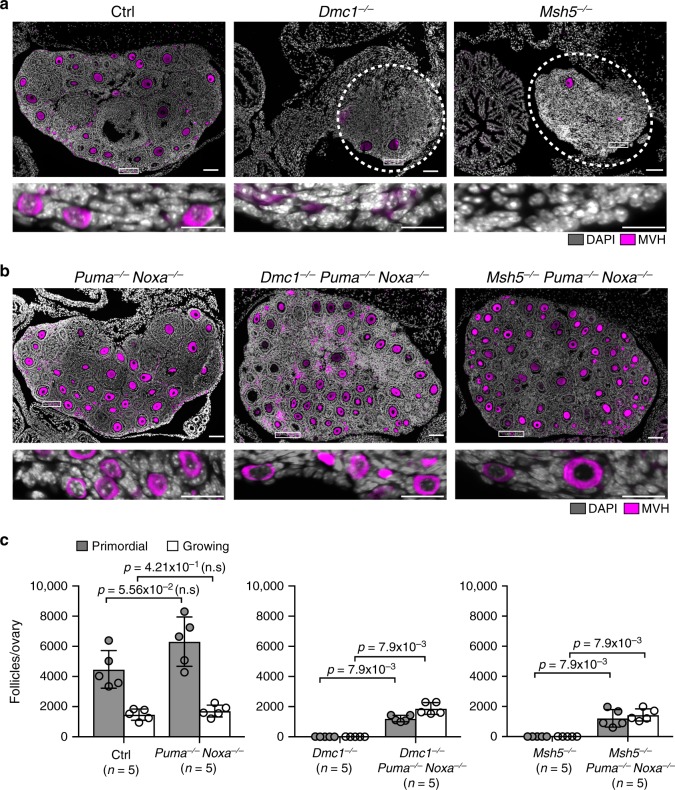
*Puma* and *Noxa* deletion rescues oocyte loss in *Dmc1* and *Msh5* nulls. **a**, **b** P21 ovary sections immunostained for oocyte marker MVH (magenta). Control (Ctrl) is *Dmc1*^+/−^ *Puma*^+/−^ *Noxa*^+/−^. White rectangles in upper panels show cortex, which is magnified in lower panels. Dotted circles outline degenerated ovaries (*n* = 5 females, *N* = 3 experimental repetitions). Scale bar in upper panels 100 μm, scale bars in micrographs 25 μm. **c** Primordial and total follicle quantitation at P21. Data are represented as mean ± standard deviation. Error bars indicate 95% confidence intervals. Two-sided Mann–Whitney test was used to calculate *p*-values.

In *Dmc1*^−/−^ and *Msh5*^−/−^ females, meiotic recombination is severely impaired resulting in persistent DNA-damage and chromosome asynapsis. DMC1 acts early in recombination, by coating resected DSBs to facilitate single-strand invasion and recombination repair^31,32^, while MSH5 acts later to stabilise double-Holliday junctions^33,34^. In *Dmc1*^−/−^ and *Msh5*^−/−^ females, persistent DSBs cause oocyte loss that is already evident at P0 (birth)^4–7^, and by P21 primordial and more advanced follicles are scarce (Fig. 1a). We examined whether P21 oocyte counts in *Dmc1*^−/−^ and *Msh5*^−/−^ females were influenced by the deletion of *Puma* and *Noxa*. Ovaries from *Dmc1*^−/−^ and *Msh5*^−/−^ females lacking either *Puma* only, or *Noxa* only, contained no primordial follicles. A few more advanced follicles were observed in these models, but not at an abundance different to that in *Dmc1*^−/−^ and *Msh5*^−/−^ females at a statistical significance of *p* < 0.05 (Supplementary Fig. 1b, c). However, *Dmc1*^−/−^ *Puma*^−/−^ *Noxa*^−/−^ and *Msh5*^−/−^ *Puma*^−/−^ *Noxa*^−/−^ P21 ovaries contained primordial as well as more developmentally advanced follicles (Fig. 1b). Total oocyte counts in *Dmc1*^−/−^ *Puma*^−/−^ *Noxa*^−/−^ and *Msh5*^−/−^ *Puma*^−/−^ *Noxa*^−/−^ females were 38% and 34% of those observed in *Puma*^−/−^ *Noxa*^−/−^ females, respectively (Fig. 1c). Combined immunostaining for the chromosome axis protein SYCP3 and the DSB marker RPA2 confirmed that at P0 meiotic DNA damage was present in *Dmc1*^−/−^ *Puma*^−/−^ *Noxa*^−/−^ and *Msh5*^−/−^ *Puma*^−/−^ *Noxa*^−/−^ oocytes (Supplementary Fig. 2a). At P0 the mean RPA2 count was similar between *Dmc1*^−/−^ *Puma*^−/−^ *Noxa*^−/−^ and *Dmc1*^−/−^, and between *Msh5*^−/−^ *Puma*^−/−^ *Noxa*^−/−^ and *Msh5*^−/−^ oocytes, at this age (Supplementary Fig. 2b). Oocyte survival is therefore due to checkpoint attenuation, rather than activation of an alternative DSB-repair pathway. We conclude that *Puma* and *Noxa* co-operate in the downstream output of the oocyte DNA-damage checkpoint.

### *Bax* deletion rescues oocyte loss in *Dmc1* and *Msh5* nulls

**Fig. 2:**
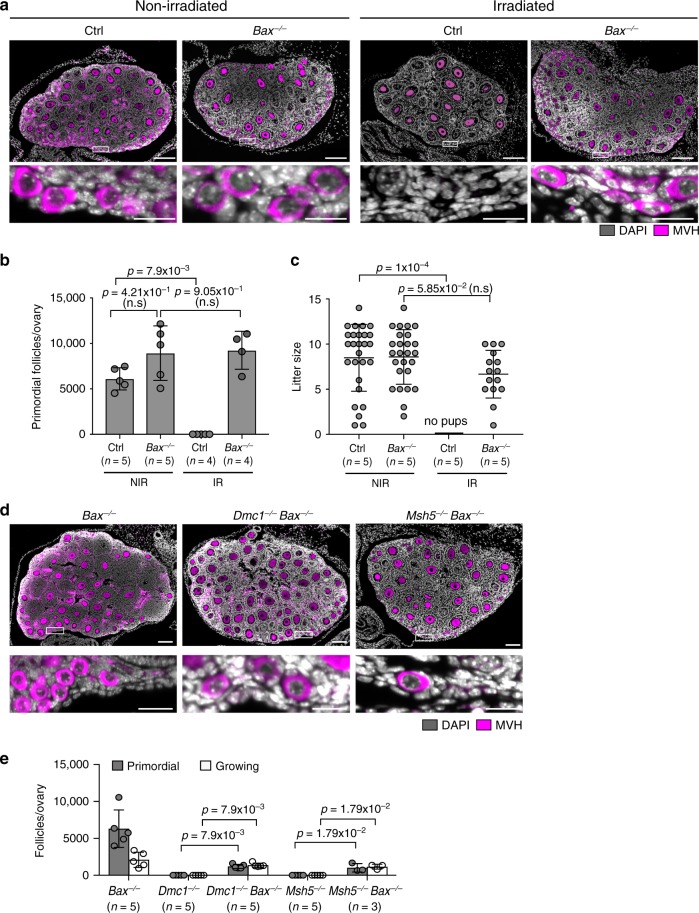
*Bax* deletion rescues oocyte loss in *Dmc1* and *Msh5* nulls. **a** P10 ovary sections immunostained for oocyte marker MVH (magenta), harvested following 0.45 Gy IR-exposure at P5 (versus non-irradiated; NIR). Control (Ctrl) is *Bax*^+/−^. White rectangles in upper panels show cortex, which is magnified in lower panels. **b** Primordial follicle quantitation at P10. Data are represented as mean ± standard deviation. Error bars indicate 95% confidence intervals. Two-sided Mann–Whitney test was used to calculate *p*-values. **c** Litter sizes from 6-week-old NIR or IR-treated females (five mating pairs for each group). Each circle represents a litter. Data are represented as mean ± standard deviation. Error bars indicate 95% confidence intervals. Two-sided Mann–Whitney test was used to calculate *p*-values. **d** P21 ovary sections immunostained for oocyte marker MVH (magenta). **e** Primordial and total follicle quantitation at P21. Note that *Dmc1*^−/−^ and *Msh5*^−/−^ data is the same as that used in Fig. 1. In all panels, scale bar 100 μm, scale bars in micrographs 25 μm. Data are represented as mean ± standard deviation. Error bars indicate 95% confidence intervals. Two-sided Mann–Whitney test was used to calculate *p*-values.

We tested whether *Bax* drives oocyte elimination in response to IR or persistent meiotic DNA damage. *Bax* deletion in non-irradiated females resulted in a slightly higher number of primordial follicles compared to their wild-type counterparts, although the difference was not statistically significant at *p* < 0.05. However, *Bax* deletion fully rescued IR-induced oocyte loss (Fig. 2a, b). Primordial follicle counts in irradiated *Bax*^−/−^ females were equivalent to those in non-irradiated *Bax*^−/−^ mice (Fig. 2b). In addition, while irradiated control females were sterile, irradiated *Bax*^−/−^ females were fertile. The mean litter size from irradiated *Bax*^−/−^ mothers was slightly lower but not significantly different (at *p* < 0.05) to that from non-irradiated *Bax*^−/−^ and *Bax*^+/−^ mothers (Fig. 2c).

The deletion of *Bax* also enabled oocyte survival in *Dmc1*^−/−^ and *Msh5*^−/−^ females (Fig. 2d). At P21, primordial follicles were present, and the total oocyte count in *Dmc1*^−/−^ *Bax*^−/−^ and *Msh5*^−/−^ *Bax*^−/−^ females was 31% and 26% of that in *Bax*^−/−^ females, respectively (Fig. 2e). We conclude that like PUMA and NOXA, BAX contributes to oocyte loss in response to both IR and persistent meiotic DNA damage. In addition, our finding that *Bax* deletion fully rescues IR- but not meiotic DSB-induced oocyte elimination demonstrates that the effector responses to these two sources of DNA damage are not identical.

### DSB markers eventually diminish in rescued oocytes

We established whether meiotic DSBs are eventually repaired in rescued oocytes. To do this, we examined whether RPA2 foci persisted in oocytes later in development, at P7. For this analysis we focused on the *Dmc1*^−/−^ *Puma*^−/−^ *Noxa*^−/−^ model, in which RPA2 foci were particularly abundant at P0 (Supplementary Fig. 2a, b). Since axis proteins including SYCP3 disappear postnatally, we identified oocytes using the germ cell-specific marker, GCNA (Supplementary Fig. 2a). The mean RPA2 count at this age was 10-fold lower than that at P0 (Supplementary Fig. 2b). Since oocyte rescue in *Dmc1*^−/−^ *Puma*^−/−^ *Noxa*^−/−^ females was incomplete, with one-third of the oocyte pool escaping elimination, this reduction may have reflected preferential survival of oocytes with fewer DSBs. However, this hypothesis could not fully explain the RPA2 decrease: all *Dmc1*^−/−^ *Puma*^−/−^ *Noxa*^−/−^ oocytes contained more than 100 RPA2 foci at P0, while more than one half exhibited an RPA2 count of zero at P7 (Supplementary Fig. 2b). We also observed *Dmc1*^−/−^ *Puma*^−/−^ *Noxa*^−/−^ oocytes at P7 containing few or no foci for a second DSB marker, RAD51 (Supplementary Fig. 2d, e). We conclude that in a cohort of surviving *Dmc1*^−/−^ *Puma*^−/−^ *Noxa*^−/−^ oocytes DSB counts diminish during maturation, possibly reflecting DSB repair.

### *Dmc1* null oocytes exhibit aberrant chromosome segregation

Our data showed that the co-deletion of *Puma* and *Noxa*, or deletion of *Bax* only, permitted survival of recombination-defective oocytes to P21. However, it was unclear whether the rescued oocytes would mature into eggs that could be fertilised. To achieve competence for fertilisation, oocytes must resume meiosis and undergo a cascade of events. These include the interaction of chromosomes with spindle microtubules, stable alignment of chromosome pairs at the spindle equator, and subsequent partitioning of homologous chromosomes, so that one member of each pair remains in the egg while the other is segregated to the polar body. We assessed each of these events, focusing on the *Dmc1*^−/−^ *Puma*^−/−^ *Noxa*^−/−^ model.

Germinal vesicle-stage oocytes persisted in *Dmc1*^−/−^ *Puma*^−/−^ *Noxa*^−/−^ females at 8–10 weeks, albeit at reduced numbers relative to control *Dmc1*^+/−^ *Puma*^+/−^ *Noxa*^+/−^ females (Supplementary Fig. 3a), as expected given findings at P21 (Fig. 1c). However, in contrast to the control oocytes, which are surrounded by easily dissociable cumulus cells^35^ (Supplementary Fig. 3b; top panel), germinal vesicle-stage oocytes from *Dmc1*^−/−^ *Puma*^−/−^ *Noxa*^−/−^ females were usually surrounded by several layers of tightly-associated cumulus cells resistant to dissociation by mechanical pipetting, indicative of incomplete growth (Supplementary Fig. 3b; bottom panel). Such non-easily dissociable cumulus oocyte complexes are similar to compact cumulus cells that surround immature GV oocytes from prepubertal mice^36,37^. Thus, if oocytes from *Dmc1*^−/−^ *Puma*^−/−^ *Noxa*^−/−^ females could complete meiosis and become successfully fertilised, these events would have to rely on the characteristic ovarian follicle pool persisting in the absence of DMC1, PUMA, and NOXA.

**Fig. 3:**
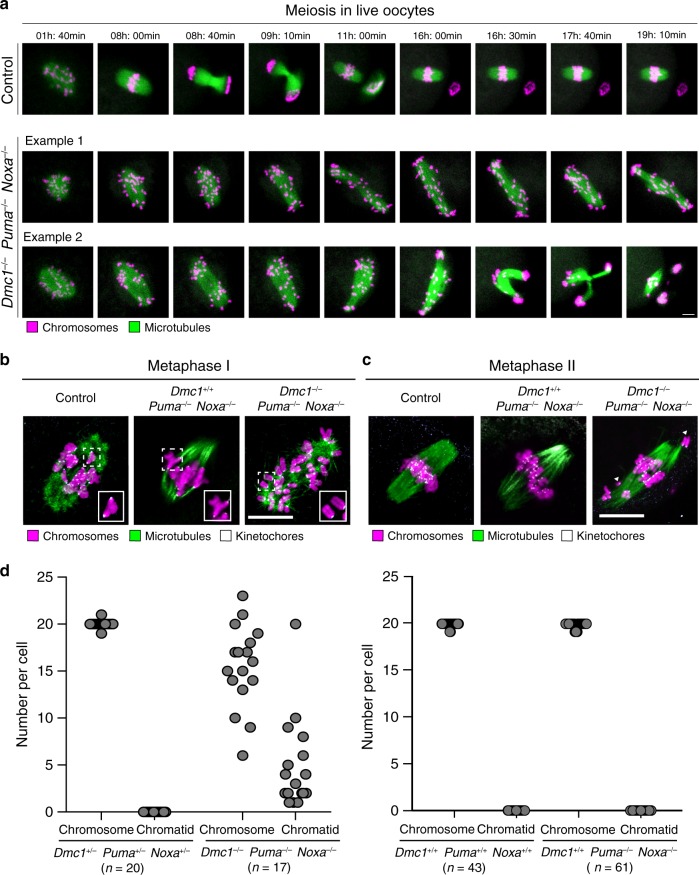
*Dmc1*^−/−^*Puma*^−/−^*Noxa*^−/−^ oocytes resume meiosis. **a** Different stages of meiosis determined from live imaging in control (*N* = 3 experimental repetitions; *n* = 7 females; *n* = 39 oocytes) and *Dmc1*^−/−^ *Puma*^−/−^ *Noxa*^−/−^ oocytes (*N* = 3 experimental repetitions; *n* = 7 females; *n* = 60 oocytes) expressing green fluorescent protein–microtubule-associated protein 4 (mEGFP–MAP4; green), and histone 2B–monomeric red fluorescent protein 1 (H2B–mRFP1; magenta). Images show *z*-projections of 21 sections, every 3 μm. Scale bar, 10 µm. Time displayed in hours:minutes. **b**, **c** Representative images of chromosomes (Hoechst, magenta), kinetochores (CREST, white), and microtubules (α-tubulin, green) in metaphase I and metaphase II (*z*-projections every 0.19 μm) cold-treated oocytes from control (*Dmc1*^+/−^ *Puma*^+/−^ *Noxa*^+/−^ or *Dmc*^+/+^ *Puma*^+/+^ *Noxa*^+/+^; *n* = 10 females), *Dmc1*^+/+^ *Puma*^−/−^ *Noxa*^−/−^ (*n* = 8 females) and *Dmc1*^−*/−*^ *Puma*^−/−^ *Noxa*^−/−^ (*n* = 2 females). Insets show a magnification of a meiosis I bivalent chromosome (left and middle) or two prematurely dissociated univalent chromosomes (right). All oocytes preserved at metaphase I had all 20 chromosomes in either the bivalent or in the univalent morphology. Scale bar, 10 µm. **d** Graph representing the number of chromosomes and chromatids per metaphase II oocyte (left graph: *N* = 2 experimental repetitions; *n* = 2 control *Dmc1*^+/−^ *Puma*^+/−^ *Noxa*^+/−^ females, *n* = 20 oocytes; *n* = 2 *Dmc1*^−/−^ *Puma*^−*/*−^ *Noxa*^−/−^ females, *n* = 17 oocytes; right graph: *N* = 2 experimental repetitions; *n* = 8 control *Dmc*^+/+^ *Puma*^+/+^ *Noxa*^*+/+*^ females, *n* = 43 oocytes; *n* = 8 *Dmc*^+/+^ *Puma*^−/−^ *Noxa*^−/−^ females, *n* = 61 oocytes).

To fully assess the developmental potential of these *Dmc1*^−/−^ *Puma*^−/−^ *Noxa*^−/−^ germinal vesicle-stage oocytes, we fluorescently labelled their chromosomes (H2B-mRFP) and microtubules (MAP4-mEGFP) and followed homologue behaviour in real-time. Morphological analysis of oocytes after 19–21 h in culture showed that polar body extrusion occurred in the majority of oocytes from control females. In contrast, polar body extrusion took place in only 41% of *Dmc1*^−/−^ *Puma*^−/−^ *Noxa*^−/−^ oocytes (Supplementary Fig. 3c), and was delayed by a mean of 7 h, suggesting a defect in chromosome segregation. Consistent with this finding, live-cell imaging of *Dmc1*^−/−^ *Puma*^−/−^ *Noxa*^−/−^ oocytes showed severe chromosome congression defects. The equatorial metaphase configuration typical of controls was never achieved, and instead homologous chromosomes were scattered along the spindle length (Fig. 3a and Supplementary Movie 1). As meiosis progressed, *Dmc1*^−/−^ *Puma*^−/−^ *Noxa*^−/−^ oocytes exhibited an elevation in spindle length and volume relative to controls (Supplementary Fig. 3d, e). Of those oocytes that extruded a polar body, 71% exhibited tripolar anaphase (Fig. 3a and Supplementary Movie 1). Kinetochore immunostaining at metaphase I revealed biorientated chromosome bivalents in control and in *Dmc1*^+/+^ *Puma*^−/−^ *Noxa*^−/−^ females. However, in *Dmc1*^−/−^ *Puma*^−/−^ *Noxa*^−/−^ females, all chromosomes were present as univalents and hence crossovers were never observed (Fig. 3b; see legend for quantitation). Thus, while DMC1 may not be required for repair of meiotic DSBs in maturing oocytes (Supplementary Fig. 2a, b), it is essential to ensure that such repair produces crossovers. Crossover defects are a well-established cause of chromosome mis-segregation in oocytes^38^ and therefore likely explain the segregation defects we observe in *Dmc1*^−/−^ *Puma*^−/−^ *Noxa*^−/−^ females.

We assayed chromosome behaviour in *Dmc1*^−/−^ *Puma*^−/−^ *Noxa*^−/−^ oocytes that had achieved polar body extrusion. Triple mutant metaphase II oocytes assembled meiotic spindles, and the majority of chromosomes achieved alignment at the metaphase plate (Fig. 3c, right panel). This finding suggests that the chromosome congression defect observed at metaphase I is likely a consequence of unpaired univalents, rather than reflecting an intrinsic inability of *Dmc1*^−/−^ *Puma*^−/−^ *Noxa*^−/−^ oocytes to achieve chromosome alignment. All the metaphase II oocytes were chromosomally abnormal, with losses or gains of whole chromosomes, as well as the presence of single chromatids resulting from premature sister separation at anaphase I (Fig. 3d, Supplementary Fig. 3f, left panels, and Supplementary Table 1). These defects are a consequence of *Dmc1* loss, as most control and *Dmc1*^+/+^ *Puma*^−/−^ *Noxa*^−/−^ oocytes cultured under these conditions were euploid (Fig. 3d, Supplementary Fig. 3f, right panels, and Supplementary Table 1). We conclude that in the absence of *Puma* and *Noxa*, recombination-defective oocytes not only escape apoptosis, but also attempt to segregate their chromosomes, generating MII oocytes with aberrant chromosome complements.

### *Dmc1* null oocytes can support fertilisation

Two defining events of fertilisation are extrusion of a second polar body and formation of a distinct male and female pronucleus within the resulting zygote. We assessed using live imaging whether these events took place following natural (non-superovulated) matings between *Dmc1*^−/−^ *Puma*^−/−^ *Noxa*^−/−^ mothers and wild type fathers. The mean number of zygotes derived from *Dmc1*^−/−^ *Puma*^−/−^ *Noxa*^−/−^ mothers was not significantly different at *p* < 0.05 to that derived from control mothers (Supplementary Fig. 4a). We obtained similar results from crosses between *Dmc1*^−/−^ *Bax*^−/−^ mothers and wild type fathers (Supplementary Fig. 4b). Thus, *Dmc1*^−/−^ *Puma*^−/−^ *Noxa*^−/−^ and *Dmc1*^−/−^ *Bax*^−/−^ eggs can support fertilisation.

**Fig. 4:**
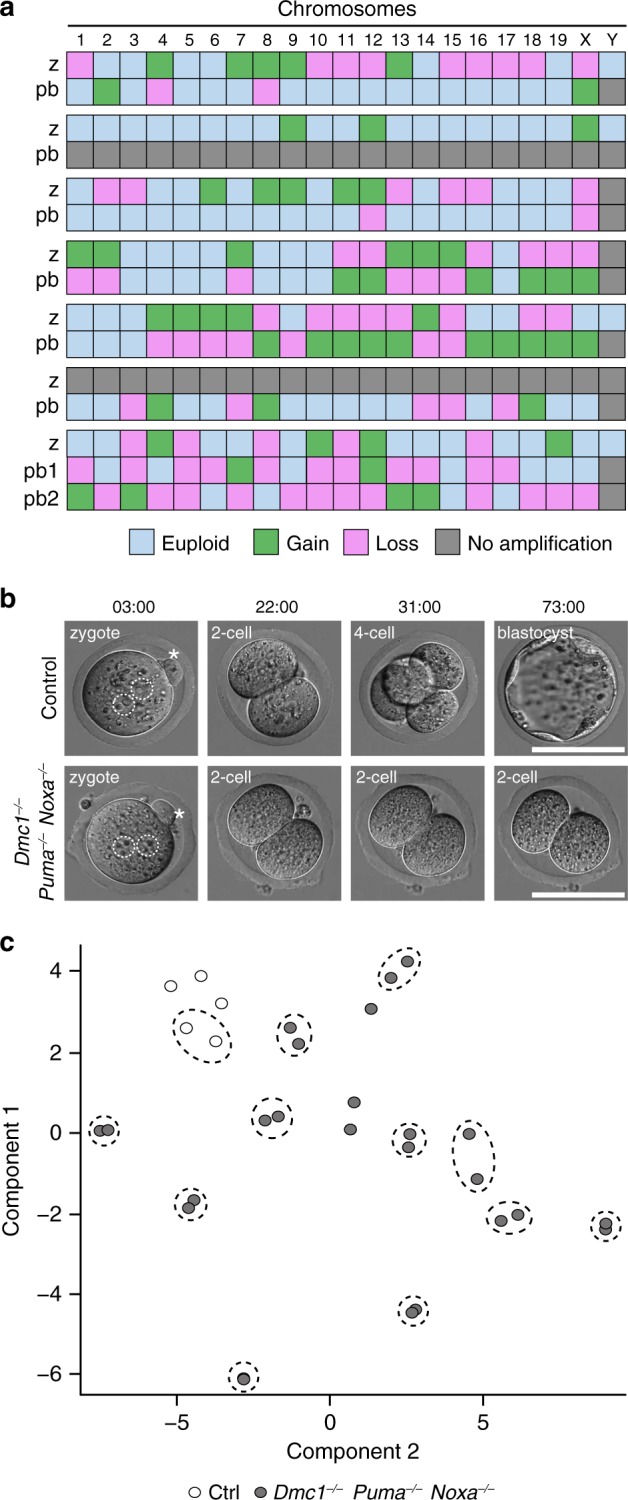
*Dmc1*^−/−^*Puma*^−/−^*Noxa*^−/−^ females generate embryos. **a** Heatmap showing chromosome gains and losses in *Dmc1*^−/−^ *Puma*^−/−^ *Noxa*^−/−^ zygotes and their matched polar body, showing multiple reciprocal chromosomal aneuploidies. **b** Development of embryos from control (*n* = 3 females, *n* = 48 zygotes, *N* = 2 experimental repetitions) and *Dmc1*^−/−^ *Puma*^−/−^ *Noxa*^−/−^ females (*n* = 3 females, *n* = 37 zygotes, *N* = 2 experimental repetitions). Dotted circles outline paternal and maternal pro-nuclei. Asterisks indicate second polar body. Time shows hours from zygote collection. Scale bar 100 µM. **c** *t*-SNE plot of single-cell RNA-seq analysis of control and *Dmc1*^−/−^ *Puma*^−/−^ *Noxa*^−/−^ blastomeres. Dotted circles represent pairs of blastomeres derived from the same embryo. Control (Ctrl) is *Dmc1*^+/−^ *Puma*^+/−^ *Noxa*^+/−^.

We used low-coverage whole-genome sequencing (less than 0.01× coverage) to examine the chromosome complement of zygotes derived using *Dmc1*^−/−^ *Puma*^−/−^ *Noxa*^−/−^ eggs and their associated polar bodies. As predicted from the striking meiotic mis-segregation phenotypes, all *Dmc1*^−/−^ *Puma*^−/−^ *Noxa*^−/−^ zygotes displayed complex aneuploidy, harbouring gains and losses affecting three or more chromosomes (Fig. 4a and Supplementary Fig. 4c). All of their associated polar bodies were also abnormal, confirming an oocyte meiotic defect with a catastrophic impact on chromosome segregation. When comparing aneuploidies detected in zygotes and corresponding polar bodies, a high degree of reciprocity was observed. For example, most chromosomal losses in polar bodies derived from *Dmc1*^−/−^ *Puma*^−/−^ *Noxa*^−/−^ mutant oocytes were associated with respective gains of material from the aneuploid chromosome in the corresponding zygote, indicating that the chromosome lost from the polar body had been retained by the oocyte. Multiple aneuploidies were present in all cases without a preference for particular chromosomes (Fig. 4a and Supplementary Fig. 4c). In contrast, all zygotes and polar bodies derived from control eggs were euploid (*n* = 9 zygotes; *n* = 9 polar bodies).

Despite the presence of aneuploidy, zygotes derived from *Dmc1*^−/−^ *Puma*^−/−^ *Noxa*^−/−^ mothers completed the first cleavage division (Fig. 4b). Live imaging revealed that the timing of cleavage relative to that of pronuclear fading was similar to that in controls (Supplementary Fig. 4d and Supplementary Movie 2). However, while embryos from control mothers continued to cleave, all those from *Dmc1*^−/−^ *Puma*^−/−^ *Noxa*^−/−^ mothers arrested at the two-cell stage (Fig. 4b). Furthermore, *Dmc1*^−/−^ *Puma*^−/−^ *Noxa*^−/−^ females produced no offspring (*n* = 4 mating pairs). In mice, the first cleavage relies on maternal products, while development beyond the two-cell stage requires the activation of embryonic transcription^39–42^. We assayed transcription in blastomeres from late two-cell embryos using single-cell RNA-sequencing (RNA-seq). Unsupervised *t*-distributed stochastic neighbour embedding (*t*-SNE) analysis showed that embryos derived from *Dmc1*^−/−^ *Puma*^−/−^ *Noxa*^−/−^ mothers did not cluster with those from control mothers (Fig. 4c). Furthermore, there was considerable transcriptional variability between individual *Dmc1*^−/−^ *Puma*^−/−^ *Noxa*^−/−^-derived embryos, presumably resulting from their distinct aneuploid chromosome complements. Arrest at the two-cell stage in embryos derived from *Dmc1*^−/−^ *Puma*^−/−^ *Noxa*^−/−^ mothers is therefore associated with chaotic gene expression.

### Distinct apoptotic effectors operate in *Spo11* nulls

Oocyte elimination occurs not only when programmed meiotic DSBs persist, but also when they are not formed. For example, *Spo11*^−/−^ females lack programmed DSBs, and this causes chromosome asynapsis and loss of oocytes around birth. Oocyte elimination in *Spo11*^–/–^ females was attributed to a distinct, HORMAD1/2-dependent synapsis checkpoint^15,16^, and to meiotic silencing^12^, the inactivation of genes on asynapsed chromosomes. However, *Spo11*^−/−^ oocytes exhibit markers of spontaneous DNA damage^19^, which could cause apoptosis via the DNA-damage checkpoint^18^. We therefore examined the effect of co-deleting *Puma* and *Noxa*, or deleting *Bax* alone, on oocyte loss in *Spo11*^−/−^ females. Interestingly, P21 primordial and total oocyte counts in *Spo11*^−/−^ *Puma*^−/−^ *Noxa*^−/−^ and *Spo11*^−/−^ *Bax*^−/−^ females were similar to those in *Spo11*^−/−^ females (Supplementary Fig. 5a, b). Thus, oocyte elimination in *Spo11*^−/−^ females is independent of *Puma*, *Noxa*, and *Bax*. We conclude that the apoptotic effectors operating in *Spo11*^−/−^ oocytes are genetically separable from those operating in *Dmc1*^−/−^ and *Msh5*^−/−^ oocytes.

## Discussion

**Fig. 5:**
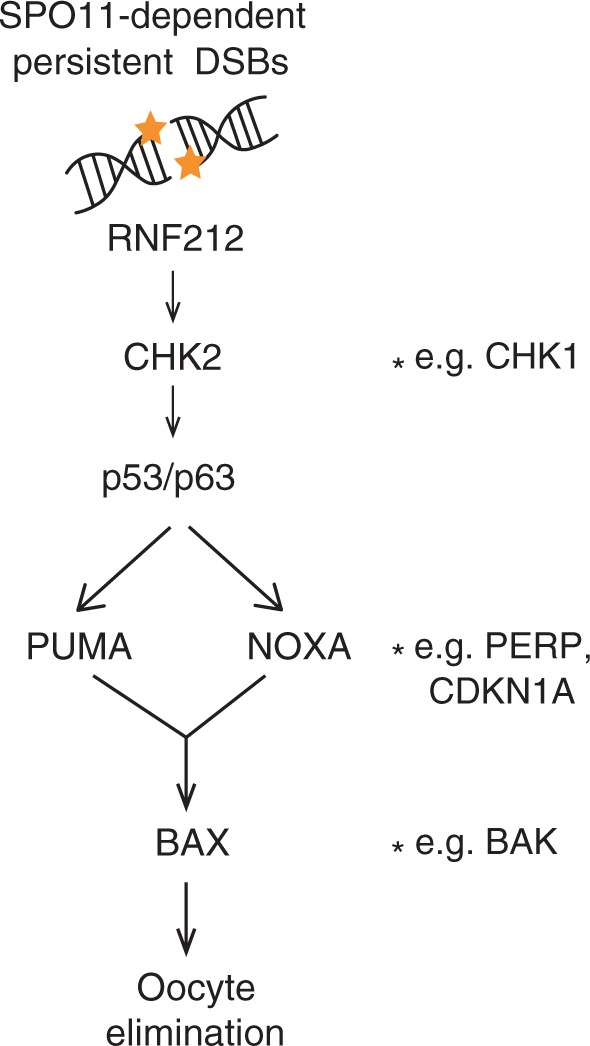
Role of PUMA NOXA and BAX in the oocyte DNA-damage checkpoint. Asterisks represent additional checkpoint effectors that may contribute to the checkpoint.

Here we identify a critical role for the BCL-2 apoptotic pathway in the mammalian oocyte DNA-damage checkpoint (Fig. 5). Integrating our work with others’, we propose that residual meiotic DNA damage, repair of which is prevented during late prophase I by RNF212, activates CHK2, thereafter inducing PUMA/NOXA/BAX-dependent apoptosis. In *Spo11*^−/−^ oocytes, chromosome asynapsis and/or spontaneous DNA damage signals via RNF212-stabilised HORMAD1/2 and CHK2, to distinct apoptotic effectors. The DNA-damage checkpoint no doubt comprises additional components (Fig. 5), because *Puma*/*Noxa* or *Bax* deletion, like *Chk2* deletion^18,43^, does not restore oocyte numbers in DSB-repair mutants to wild type levels. Furthermore, we show that the oocyte responses to IR and meiotic DSBs are not exactly the same. *Bax* deletion fully rescues IR—but not persistent meiotic DSB-induced oocyte loss. Coexistence of multiple checkpoints may ensure a robust response to the wide variety of chromosomal defects that can arise during the protracted length of female prophase I. The findings raise the possibility that *Bax* inhibitors, in addition to *Puma*/*Noxa* inhibitors^24^, may be of utility in premature ovarian failure treatment and fertility preservation in women undergoing cancer therapy.

Our data support existing evidence^24,43^ that in instances of compromised checkpoint activity, surviving oocytes exhibit some capacity to repair SPO11- or IR-induced DSBs. In *Dmc1*^−/−^ oocytes DSB focus counts diminish during prophase I exit, concomitant with disappearance of the axial element and HORMAD1, orthologues of which are negative regulators of inter-sister repair^44–47^. This point in oogenesis may mark a switch from a meiotic to a non-meiotic mechanism of DSB repair. Such repair may be beneficial in wild type oogenesis, where small numbers of lingering DSBs are insufficient to trigger oocyte elimination^18^, but could compromise embryonic viability. Deciphering whether DSB repair results in an elevated mutation frequency will be essential if *Puma, Noxa*, and *Bax* inhibitors are to be used in a clinical context.

## Methods

### Mice and animal irradiation

All animals were maintained with appropriate care according to the United Kingdom Animal Scientific Procedures Act 1986 and the ethics guidelines of the National Institute for Medical Research and Francis Crick Institute. Mice were housed in individually ventilated cages with free access to water and food. All studies were approved by local ethical review and UK Home Office. Genetically modified models are previously published: *Puma*^48^, *Noxa*^48^, and *Spo11*^2^ were maintained on C57BL/6 background; *Dmc1*^4^ and *Msh5*^6^ were maintained on MF1 background, *Bax* mice^49^ were obtained from the Jackson Labs and are maintained on a mixed C57BL/6-129 background. Littermate controls were used where possible. For the irradiation experiment, P5 female pups were exposed to a single dose of ionising radiation (0.45 Gy) in a ^137^cesium irradiator.

### Ovary section and surface spread immunofluorescence

Ovaries collected at P10 or P21 were fixed in 4% paraformaldehyde (PFA) overnight at room temperature, washed in phosphate-buffered saline (PBS), embedded in paraffin, and serially sectioned at 6 μm. For ovary section immunofluorescence, slides were deparaffinised and re-hydrated using xylene and an ethanol series, followed by antigen retrieval for 30 min in 0.1 M of sodium citrate buffer. Sections were blocked for 15 min in 5% bovine serum (PBS/Tween 20) and incubated at room temperature for 1 h with a germ cell marker (rabbit anti-MVH, 1:100, ab-13840 Abcam), followed by an incubation with secondary antibody (Alexa Fluor® 488, 1:200) and DAPI for 1 h at room temperature. Slides were mounted with Vectashield (Thermo-Fisher). Images were obtained using Olympus VS120 Slide Scanner with a U-HGLGPS mercury lamp. An Olympus UPlanApo 20×/1.35 NA objective was used. Images were captured using 2/3″ CCD camera and analysed using OlyVia Olympus software. For immunofluorescence, oocytes were treated with 0.05% Triton X-100 in water at room temperature for 10 min, and fixed in 2% PFA, 0.02% sodium dodecyl sulfate in PBS at room temperature for 1 h, washed in water, and air-dried. Slides were blocked in 0.15% bovine serum albumin, 0.1% Tween 20 in PBS at room temperature for 1 h and incubated with primary antibodies in a humidified chamber at 37 °C overnight. Antibodies used were SYCP3 (ab-15092; Abcam), GCNA (ab-82527, Abcam), RPA32 (ab-10359; Abcam), and RAD51 (PC130 Calbiochem), used at 1:100, 1:50, 1:50, and 1:100, respectively^50^. Imaging was performed using an Olympus IX70 inverted microscope with a 100-W mercury arc lamp. An Olympus UPlanApo 100×/1.35 NA oil-immersion objective was used. Images were captured by using a DeltaVision RT computer-assisted Photometrics CoolSnap HQ CCD camera with an ICX285 Progressive scan CCD image sensor. Fiji software was used to process 8- or 16-bit (512 × 512 or 1024 × 1024 pixels) captured images.

### Follicle quantification and fertility testing

For follicle quantification, every sixth section was examined for the presence of primordial, primary, secondary, and antral follicles^51^. One ovary per animal was used. Graphs and statistical analysis were performed with GraphPad Prism5. 6–8-week-old females were mated to proven fertile wild type males. Litter sizes were determined by counting pups on the day of birth.

### In-vitro culture of mouse oocytes

Oocytes were collected from ovaries of 8–10-week-old mice and cultured at 37 °C under mineral oil in homemade M2 medium or M16 medium (Sigma; MR-016), supplemented with 250 µM dbcAMP (Sigma; D0627) to maintain prophase arrest. To trigger the resumption of meiosis, oocytes were released into dbcAMP-free medium.

### Construct expression and live cell confocal microscopy

Capped mRNA was synthesised with T7 RNA polymerase (mMessage mMachine Kit Ambion), precipitated with isopropanol, and dissolved in 6 μl of RNase-free water. The following constructs were used: pGEMHE-EGFP-*MAP4* to label microtubules and pGEMHE-*H2B*-mRFP to visualise the chromosomes^52^. Quantitative microinjection was performed in a homemade chamber^37^. In brief, 10–15 oocytes were placed in a “microinjection slit” formed by separating two coverslips with a 100 μm thick piece of double stick tape. The oocytes were injected with 10 pl of mRNA. After injection of mRNA, the oocytes were immediately recovered from the chamber and the procedure was repeated for the remaining cells. Following the microinjection, the oocytes were incubated for 3 h at 37 °C to express the fluorescently-labelled proteins. Thereafter, the oocytes were release into dbcAMP-free medium and imaged. Confocal images of live oocytes were acquired using Zeiss LSM800 microscope at 37.5 °C. Oocytes were imaged in M2 medium under oil using a 40× C-Apochromat 1.2 NA water-immersion objective. The samples were imaged at a temporal resolution of 10 min and optical slice thickness of 3 μm, covering 66 μm.

### AiryScan immunofluorescence microscopy in fixed oocytes

Before fixation, kinetochore-bound microtubules were selectively depolymerised by exposing the oocytes to 4 °C for 14 min. Following the cold-treatment, the dish was removed from ice and oocytes were permeabilised by a brief 10 s exposure to 0.25% Triton X-100. Oocytes were then fixed for 30 min at 37 °C in 100 mM HEPES (pH 7; titrated with KOH), 50 mM EGTA (pH 7; titrated with KOH), 2% formaldehyde (methanol-free), and 0.2% Triton X-100. Thereafter, oocytes were extracted overnight at 4 °C in PBS supplemented with 0.1% Triton X-100. All antibody incubations were performed in PBS, 3% bovine serum albumin, and 0.1% Triton X-100, either overnight at 4 °C (primary antibodies) or for 3 h at room temperature (secondary antibodies). Primary antibodies used were human ACA centromere CREST autoantibody (FZ90C-CS1058, Europa Bioproducts; 1:500) and rat anti-α-tubulin (MCA78G, Serotec; 1:1000). As secondary antibodies, Alexa Fluor488 anti-human and Alexa Fluor647 anti-rat (Thermo Fisher; 1:400) were used. DNA was stained with 5 mg/ml Hoechst 33342 (Molecular Probes).

Fixed oocytes were imaged using the AiryScan module on Zeiss LSM800 microscope equipped with 40× C-Apochromat 1.2 NA water-immersion objectives and processed post-acquisition using ZEN2. Images were acquired at a spatial resolution of 0.19–0.30 μm optical sections, covering the entire spindle.

### Embryo collection and time lapse

6–8-week-old timed mated females were used. Zygotes were collected from the oviduct at E0.5 and washed free of the cumulus cells through a brief treatment with 3 mg/ml hyaluronidase (Sigma; H4272), washed in FHM (MerckMillipore; MR-025-D) and cultured in drops of pre-equilibrated KSOM (MerckMillipore; MR-121-D) overlaid with mineral oil (Origio; ART-4008-5P). Embryos were incubated at 37 °C and 5.5% CO_2_ in an EmbryoScope+ time-lapse incubator (Vitrolife) for 1–2 days.

### Dissociation of polar body and blastomeres

Embryos were washed in FHM and the zona pellucidae removed by a brief incubation in acidic tyrode’s solution (Sigma; T1788), followed by washing in FHM containing 10% serum supplement (Origio; ART-3001) and incubation in Accutase (ThermoFisher Scientific; A1110501) for 5–7 min. Polar bodies and blastomeres were washed in PBS and transferred to 0.5 ml microfuge tubes containing 2 µl of PBS, snap-frozen and stored at −80 °C.

### Low-coverage whole genome sequencing and aneuploidy analysis

Zygotes and polar bodies were subjected to lysis and whole genome amplification using SurePlex (Illumina). The amplified DNA was used to prepare libraries using the SQK-LSK-108 kit (Oxford Nanopore Technologies) according to the manufacturer’s instructions and sequenced on the MinION device (Oxford Nanopore Technologies), with the average number of mapped reads suitable for downstream analysis being approximately 200,000 per sample. Upon the completion of the sequencing run, reads which obtained a quality score of 7 or higher (determined as the threshold by the manufacturer), were demultiplexed using the Epi2Me software (Oxford Nanopore Technologies) and saved in fastq format. Adapters and barcodes were trimmed using the Porechop tools and the sequences were aligned to the *Mus musculus* genome (GRCm38_68) in Minimap2, using the default parameters^53^. BAM files were generated from SAM format, sorted and indexed in Samtools^54^. The reads were subsequently counted on a per chromosome basis in the Genome Analysis Tool Kit (Broad Institute) using the CountReads function^55^. The proportion of reads was then determined for each chromosome from the total number of aligned reads and compared to a reference set comprising data compiled from multiple karyotypically normal female and male samples. For all chromosomes in female samples, the resulting values were doubled prior to plotting them, generating the predicted copy number profiles, while for male samples only values from autosomes were transformed in this way, since X and Y chromosomes are each present in a single copy in euploid cells.

### Preparation of single-cell libraries for RNA sequencing

Cells from cleaving embryos were individually collected in 5 ul of PBSA, and used in the subsequent steps of cDNA synthesis and library construction. Single-cell cDNA was prepared using the SMART-Seq v4 Ultra Low Input RNA Kit (Cat. no. 634891, Clontech Laboratories), adhering to the manufacturer’s protocol. Amplified double-stranded cDNA samples that had passed quality control checks were subsequently used to make libraries. A minimum of 1 ng cDNA was used for the preparation of libraries, which was performed using the NexteraXT kit (Cat. no. FC -131-1096).

### RNA sequencing, read processing

32 Single-cell libraries were subjected to paired-end sequencing on the Illumina HiSEq 4000 analyser with 100 bp reads. Quality control was performed on FASTQ output files using the FastQC package^56^. Removal of adapters and low-quality bases from paired reads were performed using Trim Galore for each library, trimming of 11 base pairs from the 5′-end and 3 base pairs from the 3′-end. Quality was checked once again using FastQC, which showed mean quality values across each base for reads from each library typically being above 37, and the most frequently observed mean quality was 40 per sequence. Thus, trimmed paired-end reads were used in the subsequent analysis.

### Analysis of RNA sequencing

Reads from each library were first aligned to the mouse genome (Grcm38) using HISAT2 v2.1.0 with the supplied index (*genome_snp_tran*)^57^. Uniquely mapped reads (i.e., fragments that mapped once only to the reference genome) and with a MAPQ score >40 were retained for further analysis. These were then reverted to FASTQ format using Samtools (htslib 1.8) bam2fq prior to input for further steps. Transcript abundances were determined using Salmon (v0.11.3)^58^ in transcripts per million (TPM). Mouse transcriptome annotation was obtained from Ensembl (ftp://ftp.ensembl.org/pub/release-94/fasta/mus_musculus/cdna/Mus_musculus.GRCm38.cdna.all.fa.gz), which was used to create a quasi index.

Transcript abundances were then compiled and processed in R (v. 3.5.1). Briefly transcript abundances from samples were imported in the R environment using tximport^59^, and aggregated to gene-level abundances in TPM. Subsequent single cell analyses were performed using the Monocle package^60^.

### Reporting summary

Further information on research design is available in the Nature Research Reporting Summary linked to this article.

## Supplementary information

Supplementary Information

Description of Additional Supplementary Files

Supplementary Movie 1

Supplementary Movie 2

Reporting Summary

## Data Availability

The Next-Generation Sequencing data is available on the ArrayExpress website under “E-MTAB-8752” link. All data are available from the corresponding author upon reasonable request.
