# Supplementary material for: The BCL-2 pathway preserves mammalian genome integrity by eliminating recombination-defective oocytes
Source: Nat Commun. 2020 May 25;11:2598. doi: 10.1038/s41467-020-16441-z (PMC7248069; doi:10.1038/s41467-020-16441-z)
Supplement: Supplementary file 1 — Supplementary Information [file 41467_2020_16441_MOESM1_ESM.pdf]

Supplementary Information

E.ElInati et al.

The BCL-2 pathway preserves mammalian genome integrity by eliminating recombination-defective oocytes

Supplemetary Fig 1

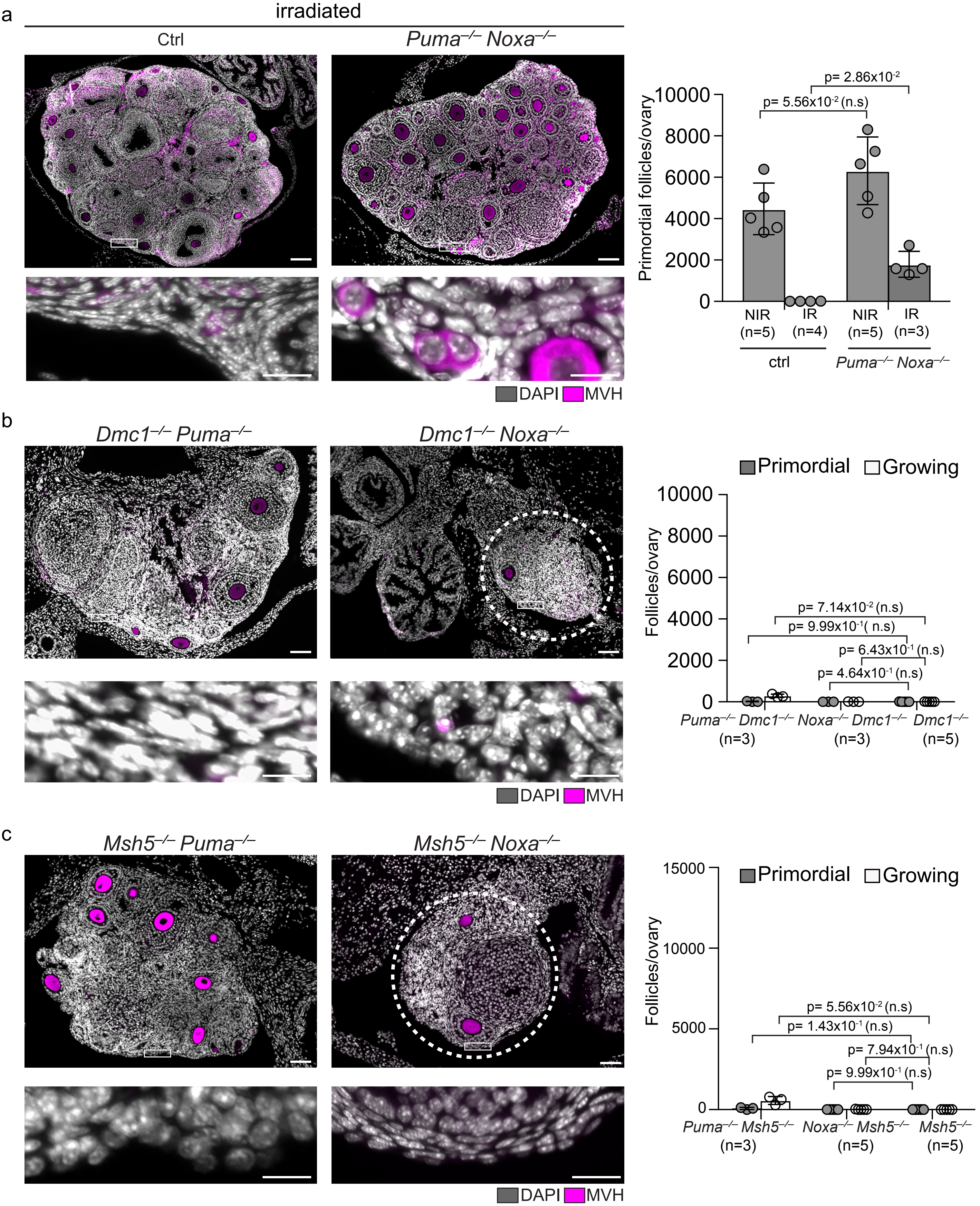

Supplementary Figure 1: Effects of *Puma* and *Noxa* on oocyte loss in response to persistent DSBs. **a**, Left: P21 ovary sections immunostained for oocyte marker MVH (magenta), harvested following 0.45Gy IR-exposure at P5. Right: Primordial follicle quantitation at P21. Control (Ctrl) is *Puma*$^{+/-}$ *Noxa*$^{+/-}$. Data are represented as mean +/- standard deviation. Error bars indicate 95% confidence intervals. Two-sided Mann-Whitney test used to calculate p-values. White rectangles in upper panels show cortex, which is magnified in lower panels. Scale bars in upper panel 100 µm, scale bars in micrographs 25 µm. **b,c**, Left: P21 ovary sections immunostained for oocyte marker MVH (magenta). Right: Primordial and total follicle quantitation at P21. Dotted circles outline degenerated ovaries. Data are represented as mean +/- standard deviation. Error bars indicate 95% confidence intervals. Two-sided Mann-Whitney test used to calculate p-values. Scale bars in upper panel 100 µm, scale bars in micrographs 25 µm.

Supplementary Fig 2

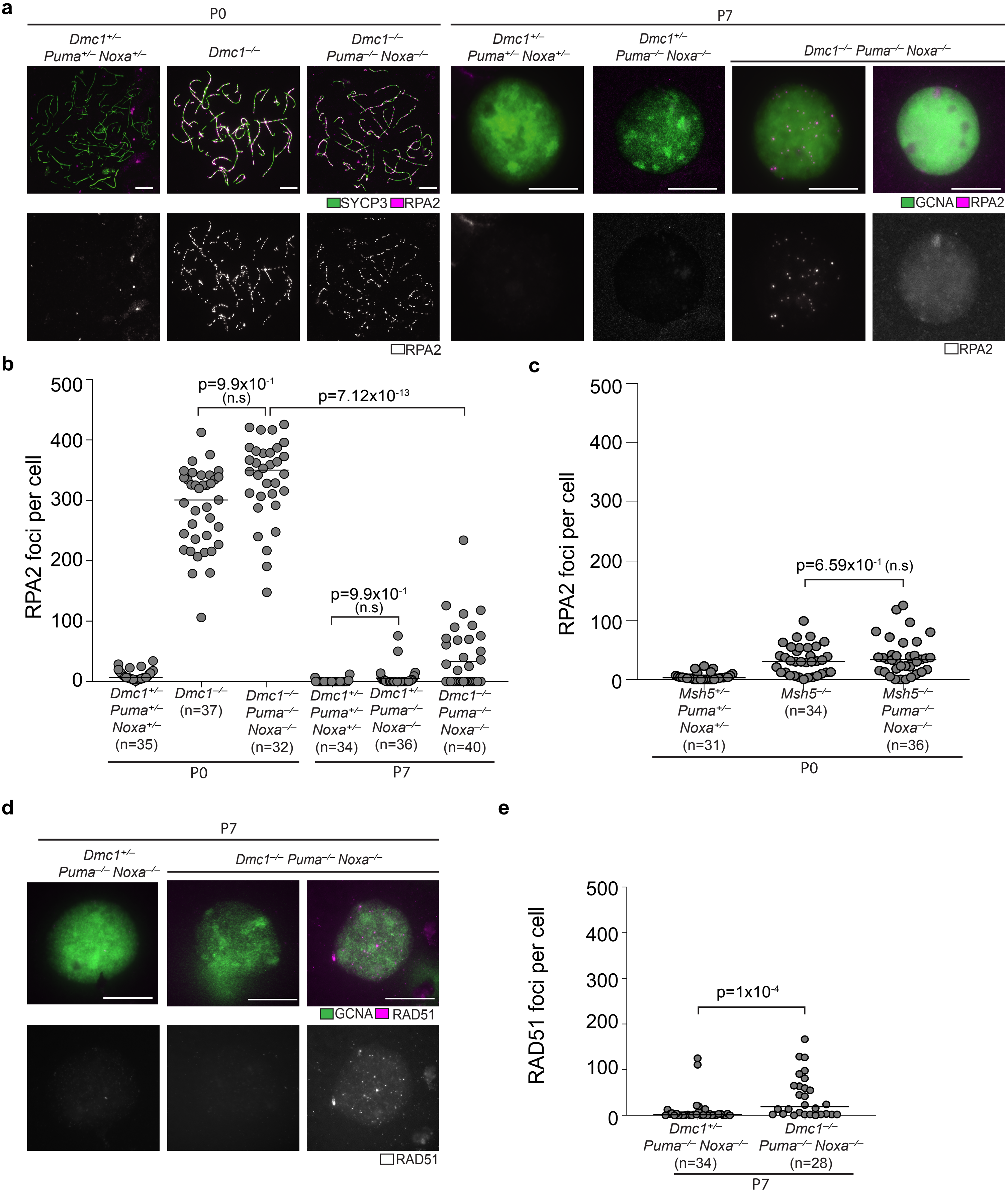

Supplementary Figure 2: DSB dynamics in control and mutant oocytes. **a**, P0 and P7 oocytes immunostained for RPA2 (magenta) and either SYCP3 (green; P0) or GCNA (green; P7). Scale bar 10 μm. **b**,**c** RPA2 focus quantitation. Horizontal line indicates the mean. For multiple comarisons, adujsted Kruskal-Wallis test used to calculate p-values otherwise a two-sided Mann-Whitney test is used with 95% confidence intervals. Control 1 (Ctrl 1) is *Dmc1*$^{+/–}$ *Puma*$^{+/–}$ *Noxa*$^{+/–}$. Control 2 (Ctrl 2) is *Msh5*$^{+/–}$ *Puma*$^{+/–}$ *Noxa*$^{+/–}$. **d,** P7 oocytes immunostained for Rad51 (magenta) and GCNA (green). Scale bar 10 μm. **e,** RAD51 focus quantitation. Horizontal line indicates the mean. Two-sided Mann-Whitney test used to calculate p-values with 95% confidence intervals.

Supplementary Fig 3

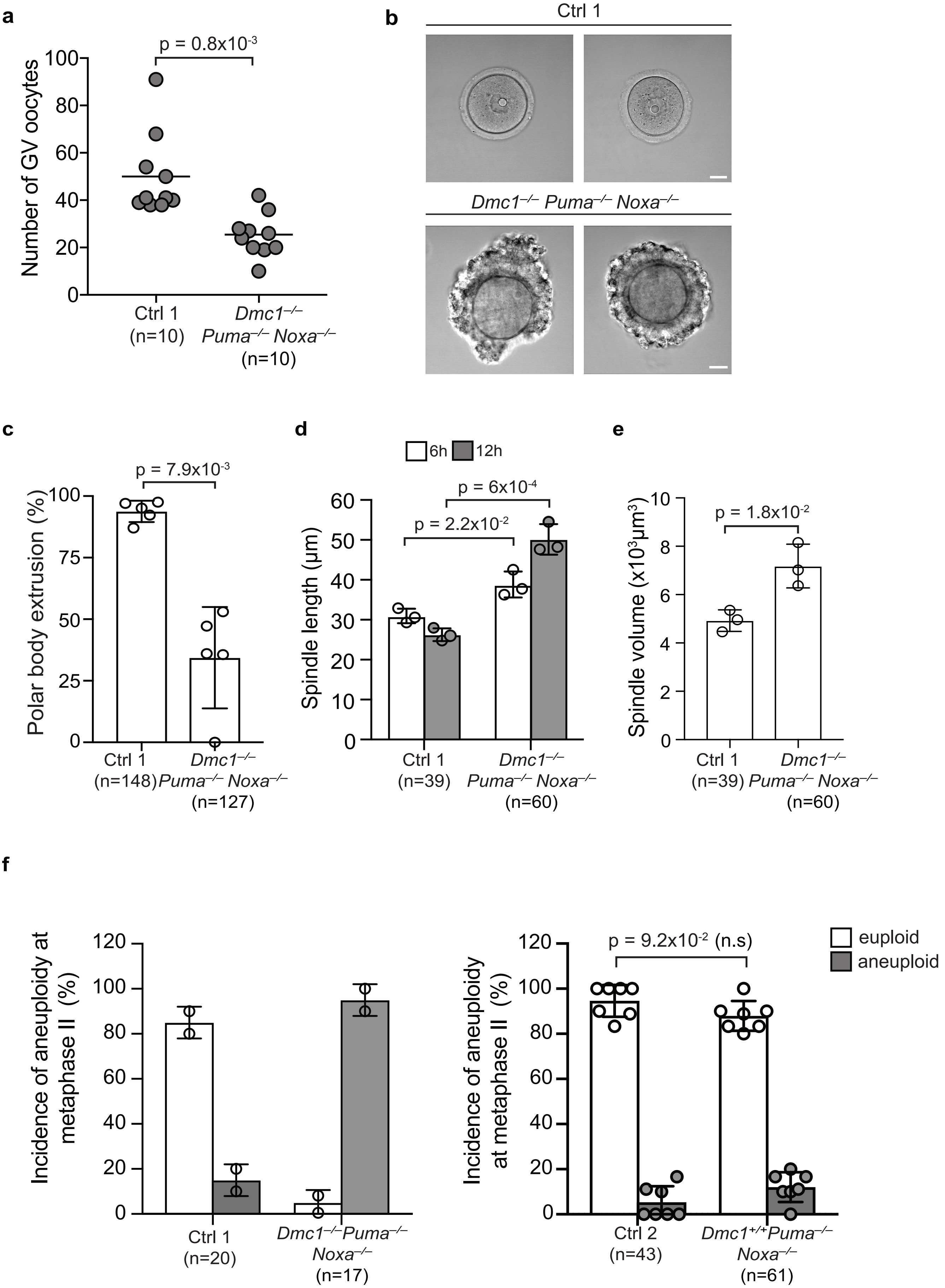

Supplementary Figure 3: Efficiency of anaphase progression. **a,** Quantification of germinal vesicle oocytes from Ctrl 1 *Dmc1*$^{+/-}$ *Puma*$^{+/-}$ *Noxa*$^{+/-}$ females (n=10 females) and *Dmc1*$^{-/-}$ *Puma*$^{-/-}$ *Noxa*$^{-/-}$ (n=10 females). N=5 experimental repetitions. **b,** Representative images of germinal vesicle oocytes after gentle stripping with a small-bore pipette, collected from the indicated genotype (N=5 repetitions, n= 10 females). Scale bar 10 µm. **c,** Percentage of oocytes exhibiting polar body extrusion in Ctrl 1 (N= 5 experimental repetitions, n=9 females; n=148 oocytes) and *Dmc1*$^{-/-}$ *Puma*$^{-/-}$ *Noxa*$^{-/-}$ oocytes (N= 5 experimental repetitions, n=9 females; n=127 oocytes). **d,** Spindle length at 6 and 12 hours post NEBD of Ctrl 1 (N=3 experimental repetitions; n=7 females; n=39 oocytes) and *Dmc1*$^{-/-}$ *Puma*$^{-/-}$ *Noxa*$^{-/-}$ oocytes (N=3 experimental repetitions; n=7 females; n=60 oocytes). **e,** Spindle volume at 6 hours post NEBD in Ctrl 1 (N=3 experimental repetitions; n= 7 females; n= 39 oocytes) and *Dmc1*$^{-/-}$ *Puma*$^{-/-}$ *Noxa*$^{-/-}$ oocytes (N=3 experimental repetitions; n= 7 females; n=60 oocytes). **f,** Incidence of aneuploidy at metaphase II in Ctrl 1 (*Dmc1*$^{+/-}$ *Puma*$^{+/-}$ *Noxa*$^{+/-}$; n=2 females, n= 20 oocytes), Ctrl 2 (*Dmc1*$^{+/+}$ *Puma*$^{+/+}$ *Noxa*$^{+/+}$; n=8 females, n=43 oocytes**)**, *Dmc1*$^{+/+}$ *Puma*$^{-/-}$ *Noxa*$^{-/-}$ *(*n=8 females, n= 61 oocytes) and *Dmc1*$^{-/-}$ *Puma*$^{-/-}$ *Noxa*$^{-/-}$ (n=2 females, n= 17 oocytes). In all diagrams, data are represented as mean +/- standard deviation. Error bars indicate 95% confidence intervals. Unpaired student two-sided t-test used to calculate p-values.

Supplementary Fig 4

a

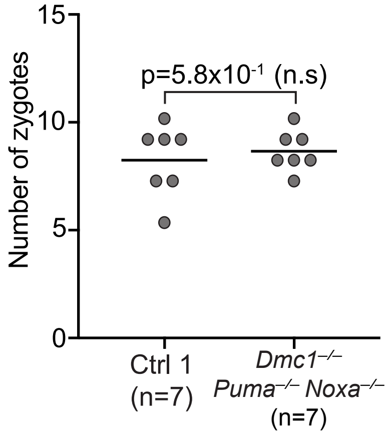

b

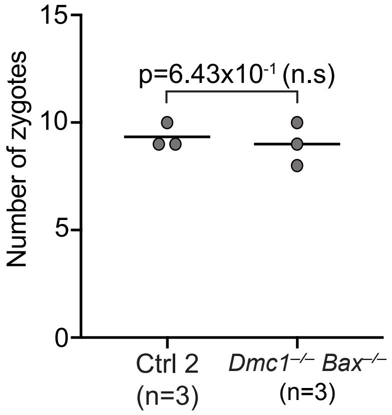

c

sample		aneuploidy
1	zygote	+1, +4 (4copies), +7, +8 (4 copies), +9, -10, -11, -12, +13, -15, -16, -17, -18, -X (0 copies),Y
	polar body	+2 (3 cht), -4 (0 cht) , -8 (0 cht), +X (3cht)
2	zygote	+9, +12 (3 or 4 copies), +X
	polar body	no amplification
3	zygote	-2, -3, +6, +8, +9, +11, +12, -13, -15, -16, -X
	polar body	-12 (1cht), -X (1cht)
4	zygote	+1, +2, +7, -11, -12, +13, +14 (4 copies), +15, -16, -18, -19, -X
	polar body	-1(1cht), -2(1cht), -7(1cht), +11(3cht), +12(4cht), -13(1cht), -14(0cht), -15(1cht), +16(3cht), +18(3cht), +19(4cht), +X(3cht)
5	zygote	+4, +5, +6, +7, -8, -10, -12, -13, +14, -15, -18, -19
	polar body	-4(0cht), -5(0cht), -6(0cht), -7(0cht), +8(4cht), -9(0cht), +10(3cht), +11(3cht), +12(3cht), +13(3cht), -14(0cht), -15(0cht), +16(3cht), +17(3cht)+18(3cht), +19(4cht), +X(3cht)
6	zygote	no amplification
	polar body	-3(0cht), +4(3cht), -7(0cht), +8(3cht), -14(0cht), -15(0cht), -17(0cht), +18(3cht)
7	zygote	-3, +4, -5, -8, +10, -11, +12(3 or 4 copies), -16, +19
	polar body	-1(0cht), -3(0cht), -5(0cht), -6(0cht), +7(4cht), -8(0cht), -10(0cht), -11(0cht), +12(3cht), -13(0cht), -14(0cht), -16(0cht), -17(0cht)
	polar body	+1(3cht), -2(0cht), +3(3cht), -4(0cht), -5(0cht), -7(0cht), -9(0cht), -10(0cht), -11(0cht), -12(0cht), +13(3cht), +14(3cht), -16(0cht), -18(0cht), -19(0cht), -X(0cht)

d

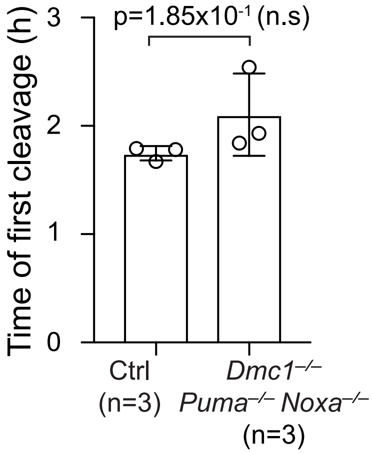

Supplementary Figure 4: Aneuploidy analysis. **a,b**, Quantification of zygotes from controls, *Dmc1*$^{-/-}$ *Puma*$^{-/-}$ *Noxa*$^{-/-}$ and *Dmc1*$^{-/-}$*Bax*$^{-/-}$ females mated to wild type males. Control 1 (Ctrl 1) is *Dmc1*$^{+/-}$ *Puma*$^{+/-}$ *Noxa*$^{+/-}$. Control 2 (Ctrl 2) is *Dmc1*$^{+/-}$ *Bax*$^{+/-}$. Horizontal line indicates the mean. Unpaired two sided student t-test used to calculate p-values with 95% confidence intervals. **c,** Table representing aneuploidy in zygotes generated from *Dmc1*$^{-/-}$ *Puma*$^{-/-}$ *Noxa*$^{-/-}$ oocytes and their respective polar bodies. "+" gain, "-" loss, "cht" chromatid. **d,** Time in hours of first cleavage division of zygotes from control (n=3 females; n=48 zygotes, N= 2 experimental repetitions) and *Dmc1*$^{-/-}$ *Puma*$^{-/-}$ *Noxa*$^{-/-}$ (n=3 females; n=37 zygotes, N= 2 experimental repetitions) females. Control (Ctrl) is *Dmc1*$^{+/-}$ *Puma*$^{+/-}$ *Noxa*$^{+/-}$. Data are represented as mean +/- standard deviation. Error bars indicate 95% confidence intervals. Two-sided unpaired student t-test used to calculate p-values.

Supplementary Fig 5

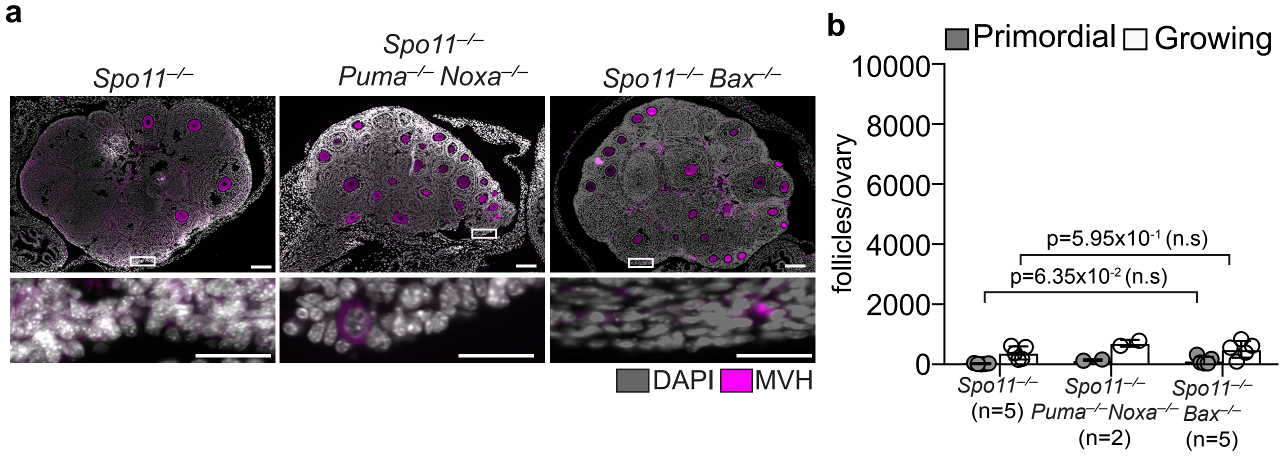

Supplementary Figure 5: Distinct apoptotic effectors result in oocyte loss in *Spo11* nulls. **a**, P21 ovary sections immunostained for oocyte marker MVH (magenta). N= 2 experimental repetitions. Scale bars in upper panel 100 µm, scale bars in micrographs 25 µm. **b**, Primordial and total follicle quantitation at P21. Data are represented as mean +/- standard deviation. Error bars indicate 95% confidence intervals. Two-sided Mann-Whitney test used to calculate p-values.

Supplementary Table 1

Ctrl 1

MII oocyte	№ of chromosomes	№ of single chromatids
1 to 18	20	0
19	21	0
20	19	0

Ctrl 2

MII oocyte	№ of chromosomes	№ of single chromatids
1 to 50	20	0
51 to 53	19	0

Dmc1$^{+/+}$Puma$^{–/–}$ Noxa$^{–/–}$

MII oocyte	№ of chromosomes	№ of single chromatids
1 to 54	20	0
55 to 61	19	0

Dmc1$^{–/–}$ Puma$^{–/–}$ Noxa$^{–/–}$

MII oocyte	№ of chromosomes	№ of single chromatids
1	13	1
2	9	2
3	17	4
4	19	8
5	15	10
6	16	2
7	6	2
8	18	9
9	10	6
10	14	2
11	17	2
12	15	20
13	21	4
14	23	1
15	14	1
16	17	5
17	20	3

Supplementary Table 1: Number of chromosomes and chromatids per oocyte. Number of chromosomes and chromatids Ctrl 1 *Dmc1*$^{+/-}$ *Puma*$^{+/-}$ *Noxa*$^{+/-}$, Ctrl 2 *Dmc1*$^{+/+}$*Puma*$^{+/+}$ *Noxa*$^{+/+}$, *Dmc1*$^{+/+}$ *Puma*$^{-/-}$ *Noxa*$^{-/-}$ and *Dmc1*$^{-/-}$ *Puma*$^{-/-}$ *Noxa*$^{-/-}$ metaphase II oocytes.
