# Supplementary material for: The BCL-2 pathway preserves mammalian genome integrity by eliminating recombination-defective oocytes
Source: Nat Commun. 2020 May 25;11:2598. doi: 10.1038/s41467-020-16441-z (PMC7248069; doi:10.1038/s41467-020-16441-z)
Supplement: Supplementary file 2 — Description of Additional Supplementary Files [file 41467_2020_16441_MOESM2_ESM.pdf]

Description of Additional Supplementary Files

File Name: Supplementary Movie 1
Description: Live imaging of control and Dmc1 $^{-/-}$ Puma $^{-/-}$ Noxa $^{-/-}$ oocytes.

File Name: Supplementary Movie 2
Description: Live imaging of embryos from control and Dmc1 $^{-/-}$ Puma $^{-/-}$ Noxa $^{-/-}$ mothers.
